# Immunohistochemical and Immunoelectron Microscopical Distribution of MEGF8 in the Mouse Central Nervous System

**DOI:** 10.3390/cells13010063

**Published:** 2023-12-28

**Authors:** Kazuhiko Nakadate, Kiyoharu Kawakami

**Affiliations:** Department of Basic Science, Educational and Research Center for Pharmacy, Meiji Pharmaceutical University, 2-522-1 Noshio, Kiyose 204-8588, Tokyo, Japan; d236953@std.my-pharm.ac.jp

**Keywords:** ubiquitin, proteasome, synapse, mitochondria, E3 ubiquitin ligase, neuron

## Abstract

Mutations in multiple epidermal growth factor-like domain 8 (MEGF8), a multidomain transmembrane protein encoded by a gene conserved across species, cause Carpenter’s syndrome, which is associated with learning disabilities, mental health issues, and left–right patterning abnormalities. MEGF8 interacts with MGRN1, a protein that functions as an E3 ubiquitin ligase and is involved in multiple physiological and pathological processes. However, the mechanism underlying the distribution of MEGF8 in the central nervous system (CNS) and its cellular and subcellular locations remain unknown. This study aimed to map MEGF8 in the mouse CNS using a new antibody. We discovered that MEGF8 was distributed in the majority of neuronal cell somata across most CNS regions. High levels of MEGF8 were expressed in the neuropils of the CNS gray matter. Immunoelectron microscopy showed that MEGF8 was present in the synapses and around the outer mitochondrial membrane. These findings show that MEGF8 is uniformly distributed throughout the mouse CNS, and its distribution indicates that it plays a substantial role in synaptic and mitochondrial functions. To the best of our knowledge, this is the first study to document MEGF8 distribution in the CNS.

## 1. Introduction

Multiple epidermal growth factor-like domain 8 (MEGF8) mutations in humans are linked to Carpenter’s syndrome, an autosomal recessive disorder characterized by heterotaxy (impaired left–right patterning), serious congenital heart defects (CHDs), duplication of the fore-axis, skeletal abnormalities, and intellectual disability [1]. Genetic screenings of mice have also revealed that MEGF8 proteins regulate both left–right patterning and cardiac morphogenesis [2,3,4]. The function of the MEGF8 homolog in *Drosophila*, dMEGF8, was recently reported [5], indicating that dMEGF8 localizes to the synapses of neuromuscular junctions and is required for proper synaptic growth. In addition, dMegf8 mutant larvae and adults exhibit severe motor coordination deficits. Furthermore, *dMegf8* mutants showed altered localization of presynaptic and postsynaptic proteins, defects in synaptic ultrastructure, and neurotransmission. Consequently, MEGF8 may play a crucial role in the in vivo developmental process in mammals and synaptic function in the adult brain. However, the physiological role of MEGF8 has yet to be clarified, as its distribution within the central nervous system (CNS) in mammals has not been analyzed.

MEGF8 has EGF-like motifs, a CUB domain, a C-type lectin domain, and a domain similar to the ligand-binding region of the common cytokine chain and attractin (Atrn) family [6]. Recently, a sequence, wherein Atrn and/or Attractin-like-1 (AtrnL1) binds to Mahognin ring finger 1 (MGRN1), was identified as a MASRPF motif, and a MEGF8 protein with a MASRPF motif was suggested [7]. MGRN1, a member of the RING family, exhibits E3 ubiquitin ligase activity and is expressed in many mouse tissues [8,9]. It interacts with GP78, an endoplasmic reticulum E3 ligase, and utilizes calmodulin as an adapter to regulate mitophagy through the proteasome. The regulation of α-tubulin ubiquitination by MGRN1 is crucial for maintaining microtubule stability and proper positioning of the mitotic spindle in mitotic cells [10], and MGRN1 expression is the highest in the brain [8,11,12]. Our previous study [13] showed that MGRN1 is expressed in the brain neurons; high MGRN1 expression was observed in gray-matter neuropils in the CNS. Furthermore, immunoelectron microscopy revealed MGRN1 expression near the neuronal presynaptic and mitochondrial membranes. 

Null mutations in *MGRN1* cause spongiform encephalopathy [9]. Although the endogenous MGRN1 substrate is unknown, the cytoplasmic tail of Atrn may be a target. Atrn, a transmembrane protein with an epidermal growth factor-like motif, CUB domain, C-type lectin domain, and a domain similar to the ligand-binding region of common cytokine chains, is abundantly expressed in human, rat, and mouse tissues, particularly in the CNS [7,12,14,15,16,17]. These domains are common in proteins involved in antigen processing and endocytosis in macrophages and dendritic cells. Atrn contains EGF-like domains and a ligand-binding area similar to that of the cytokine receptor common chain. The presence of loss-of-function mutations in Atrn, zitter, and mahogany in rats and mice leads to the display of similar neuropathological characteristics. In situ hybridization has revealed similar expression patterns of Atrn and MGRN1 mRNA in the mouse brain [9,18]. These findings support the theory that Atrn and MGRN1 help to eliminate unidentified substrates, leading to spongiform encephalopathy [9,12]. 

We previously [14] showed that Atrn is expressed in most brain neurons, particularly in large neurons, such as cortical pyramidal and cerebellar Purkinje neurons. Immunoelectron microscopy revealed the presence of Atrn in the plasma membrane of the neuron soma, dendrites, and spines as well as in the cytoplasmic membrane of the Golgi apparatus, endoplasmic reticulum, and mitochondria. It was previously assumed that the distribution of MGRN1 and Atrn was contiguous [13,14]; however, it was later discovered that there were areas where MGRN1 and Atrn existed independently from one another. This finding implies that other proteins bind to MGRN1 and initiate various activities. The homolog of Atrn, AtrnL1 [6,7], was proposed to play a role similar to Atrn and is believed to interact with MGRN1 [19]. 

The ubiquitin-proteasome system (UPS) is vital for maintaining neuronal equilibrium in the CNS [20,21,22]. When the UPS is impaired, misfolded and disassembled proteins accumulate, resulting in neurodegeneration. Loss-of-function mutations in the gene encoding the E3 ubiquitin ligase, parkin, have been linked to juvenile Parkinson’s disease [23,24,25]. Moreover, the UBE3A/E6-AP protein, another E3 ubiquitin ligase, is mutated in Angelman syndrome [26,27]. However, there is no information regarding the distribution of MEGF8 in the brain. Therefore, this study aimed to elucidate the distribution of MEGF8 and Atrn family proteins and their relationship with MGRN1. To accomplish this, we examined the distribution of MEGF8 in the mouse CNS using immunohistochemistry analysis and immunoelectron microscopy.

## 2. Materials and Methods

### 2.1. Experimental Animals

In this study, a total of twelve male ICR mice, aged 8 weeks, sourced from the Charles River Laboratory in Japan, were utilized. These animals were housed in a controlled environment with a regulated temperature and humidity, a 12 h light/dark cycle, and unlimited access to food and water. All experimental procedures were conducted in accordance with the ethical guidelines set forth by the National Institutes of Health (NIH) for the humane treatment of laboratory animals. The Meiji Pharmaceutical University Laboratory Animal Ethics Committee approved this study (No. 2706; 1 April 2020–2023), with a particular emphasis on minimizing animal suffering and reducing the number of animals used in the study.

### 2.2. Anti-MEGF8 Antibody

The procedures for generating the MEGF8 antibody have been previously detailed [13,28]. The antibody was developed using a synthetic peptide that consisted of amino acids 28–43 from the mouse MEGF8 sequence (GDCKGQRQVLREAPGF; GenBank Accession No. ACE00231.1). It was created in rabbits. We verified this sequence using BLAST. The sequence of the antigenic peptide was highly specific and remained conserved across different species, with identical rat and human MEGF8 antigenic peptide sequences. Antibodies were purified using Sepharose 4 B resin with an attached synthetic peptide (Amersham Biosciences, Buckinghamshire, UK). To determine the specificity of the affinity-purified MEGF8 antibody, it was incubated with synthetic peptides (GDCKGQRQVLREAPGF). Immunohistochemistry, Western blotting, and immunoelectron microscopy analyses were performed to analyze the supernatants.

### 2.3. Western Blotting Analysis

Three 8-week-old mice were deeply anesthetized using isoflurane (Fujifilm Wako Chemicals, Tokyo, Japan). Subsequently, all mice were perfused with 0.1 M phosphate-buffered saline solution (PBS, pH 7.4) through the left ventricle. The brain and spinal cord were promptly excised and dissected into several pieces, which were then thoroughly mixed and prepared using sodium dodecyl sulfate (SDS) buffer, to achieve a well-blended solution. Five-percent sodium dodecyl sulfate-polyacrylamide gel electrophoresis was used to separate proteins, which were then transferred onto polyvinylidene difluoride membranes. Membranes were incubated with 10% skim milk solution in PBS with 0.1% Tween 20 for 1 h at room temperature, according to the manufacturer’s instructions (Becton, Dickinson and Company, Franklin Lakes, NJ, USA). The membranes were washed and incubated with rabbit anti-MEGF8 antibody at a 1:5000 dilution for 2 h at room temperature. The membranes were washed again and incubated with horseradish peroxidase-conjugated anti-rabbit antibody at a 1:2000 dilution for 45 min at room temperature. Immunoreactive bands were determined using a chemiluminescence Western blot scanner (LI-COR C-DiGit, LI-COR Corporate, Lincoln, NE, USA). The β-tubulin primary antibody (ab52901, diluted 1:5000, Abcam, Cambridge, UK) was utilized to treat the membranes, functioning as an internal control for sample loading.

### 2.4. Immunohistochemistry

Detailed methods have been outlined previously [13,14,29]. Four 8-week-old male ICI mice were used for immunohistochemical analysis. The mice were deeply anesthetized using isoflurane (Fujifilm Wako Chemicals, Tokyo, Japan), perfused with saline, and then perfused with 4% paraformaldehyde in 0.1 M PB (pH 7.4). The brains were placed in a fixative for 24 h at 4 °C. The brains were cut into 30 μm thick sections using a cryo-microtome (Leica Microsystems in Wetzlar, Germany). Sections were incubated for 90 min in 1% hydrogen peroxide. Following the washing process, the sections were placed in a solution of 10% Block-Ace (Dainihon Seiyaku, Tokyo, Japan) in PBST and incubated for 2 h. They were then incubated for 48 h at 4 °C with an anti-MEGF8 antibody (diluted 1:3000). Next, the sections were incubated with a biotinylated goat anti-rabbit antibody (1:1000, Vector Laboratories, Inc., Burlingame, CA, USA) for 2 h, washed, and incubated with an avidin-biotin peroxidase complex (ABC kit; Vector Laboratories) for 2 h. The sections were then incubated in 0.05% 3-3-diaminobenzidine tetrahydrochloride (Dojindo, Kumamoto, Japan). All of the tissue samples were placed on slides and dehydrated using a mixture of ethanol and Lemosol, a product produced by Fujifilm Wako Pure Chemical Industries, Ltd., in Tokyo, Japan. The slides were then sealed with clear cover slips. Some sections were incubated with pre-adsorbed primary antibody. All sections were captured using a CCD camera (BZ-X700; Osaka, Keyence, Japan).

### 2.5. Immunoelectron Microscopy

Immunoelectron microscopy methods have been described previously [13,14,30,31]. Three 8-week-old ICI mice were used for immunoelectron microscopy analysis. A solution of 4% paraformaldehyde, 0.2% picric acid, and 0.05% glutaraldehyde in 0.1 M phosphate buffer (pH 7.4) was used as an ice-cold fixative, after the animals were anesthetized using isoflurane (Fujifilm Wako Chemicals, Tokyo, Japan) and perfused with physiological saline. Brains were removed and post-fixed with the same fixative for 24 h at 4 °C. The brains were washed in 0.1 M PB and sliced into 50 μm thick sections using a VT1000S microtome (Leica Microsystems). The sections were cryoprotected in 30% sucrose in 0.1 M PB and freeze-thawed. The sections were incubated in 10% normal goat serum in 0.1 M PBS for 2 h, followed by overnight incubation at 4 °C with the anti-MEGF8 antibody (1:3000). The sections were first washed and then exposed to a biotinylated anti-rabbit antibody (1:1000, Vector Laboratories) and allowed to interact with ABC and DAB. After treatment with OsO4, the sections were stained with uranyl acetate, dehydrated, and embedded in Epon-812 resin (TAAB, Berkshire, UK). Ultrathin sections (70 nm thick) were cut using a Leica EM UC6 ultramicrotome (Leica Microsystems) and imaged using a transmission electron microscope (HT7800; Hitachi, Tokyo, Japan).

### 2.6. Image Processing

The ABC-DAB reaction was utilized for the densitometric analysis of IR by means of ImageJ version 1.54, a software developed by the US National Institute of Health in Bethesda, MD. The density of the samples was evaluated on a scale of 5 distinct levels. The procedures involved in this process have been described in detail in prior publications [13,14]. The MEGF8 intensity threshold was set to twice the baseline value using ImageJ. A 5-point density scale was employed, with ++++ representing the highest density, +++ indicating higher density, ++ indicating high density, + indicating low density, and ~ indicating background density. Owing to methodological constraints, the density scale indicated the mean density of the cells and neuropil staining. Anatomical structures were identified by directly observing Nissl-stained sections, with the Paxinos and Franklin atlas serving as a guide [32]. 

Similarly, ImageJ was used for the densitometric analysis of the protein bands in the Western blot data analysis. Statistical analysis was performed using the StatView statistical software (Version 5.0, SAS Institute Inc., Cary, NC, USA). Statistical differences were analyzed using analysis of variance, and significance was set at *p* < 0.05. Data are expressed as mean ± standard deviation.

## 3. Results

### 3.1. Specificity of Antibodies

**Figure 1:**
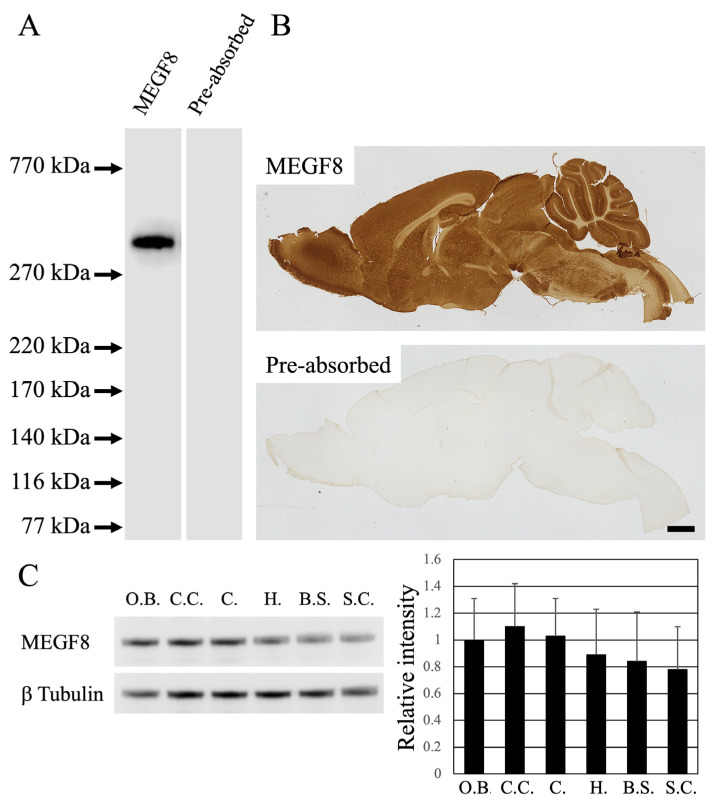
(**A**) Anti-MEGF8 antibody detected a single band of approximately 300 kDa in the mouse brain membrane fraction (MEGF8 lane). No detectable levels of immunoreactivity were observed in the Western blots generated using serum that had previously been absorbed with the synthetic peptide of the epitope (pre-absorbed lane). (**B**) Immunohistochemical staining of MEGF8 antibody in the sagittal sections of the mouse brain revealed anti-MEGF8 antibody immunoreactivity. No detectable levels of immunoreactivity were found in the serum after it was pre-absorbed with the synthetic peptide epitope (pre-absorbed). Scale bar: (**B**) = 1 mm. (**C**) Western blot analysis was used to reveal MEGF8 expression at representative sites within the CNS. The expression level of β-tubulin serves as an internal control, as demonstrated by the Western blot data (each number = 3). The densitometry analysis revealed the mean ± standard deviation, and the significance level was set at *p* < 0.05. The relative intensity is displayed as 1 = the intensity of the olfactory valve value, as shown in the data. O.B., C.C., C., H., B.S., and S.C. indicate the olfactory bulb, cerebral cortex, cerebellum, hippocampus, brain stem, and spinal cord, respectively.

The newly developed affinity-purified antibodies against MEGF8 were tested through immunoblot analysis of 8-week-old ICR mice brains (*n* = 3) (Figure 1). MEGF8 has an approximate molecular weight of 300 kDa and can be accurately identified using anti-MEFG8 antibodies (Figure 1A). The molecular mass was deemed accurate based on a gene report (NCBI reference sequence: NP_001153872.1). Furthermore, pre-incubation of the primary antibody with excess epitope peptides completely eliminated immunoreactivity (IR) (Figure 1A).

The specificity of the antibody was further validated through immunohistochemical staining by pre-incubating sections of the mouse brain with the anti-MEGF8 antibody and a synthetic immunogen peptide (*n* = 3) (Figure 1B).

Western blotting was performed to confirm the presence or absence of MEGF8 expression in representative regions within the central nervous system (*n* = 3) (Figure 1B). In this analysis, the olfactory bulb, cerebral cortex, cerebellum, hippocampus, brain stem, and spinal cord were targeted. The results showed that the levels of MEGF8 tended to be slightly higher in the cerebral cortex and cerebellum and slightly lower in the spinal cord; however, the levels were not significantly different. These results support the immunohistochemistry results, which showed strong MEGF8 expression across the regions of the central nervous system, and suggest that the antibodies are specific and useful.

### 3.2. Overview of MEGF8 Distribution

**Figure 2:**
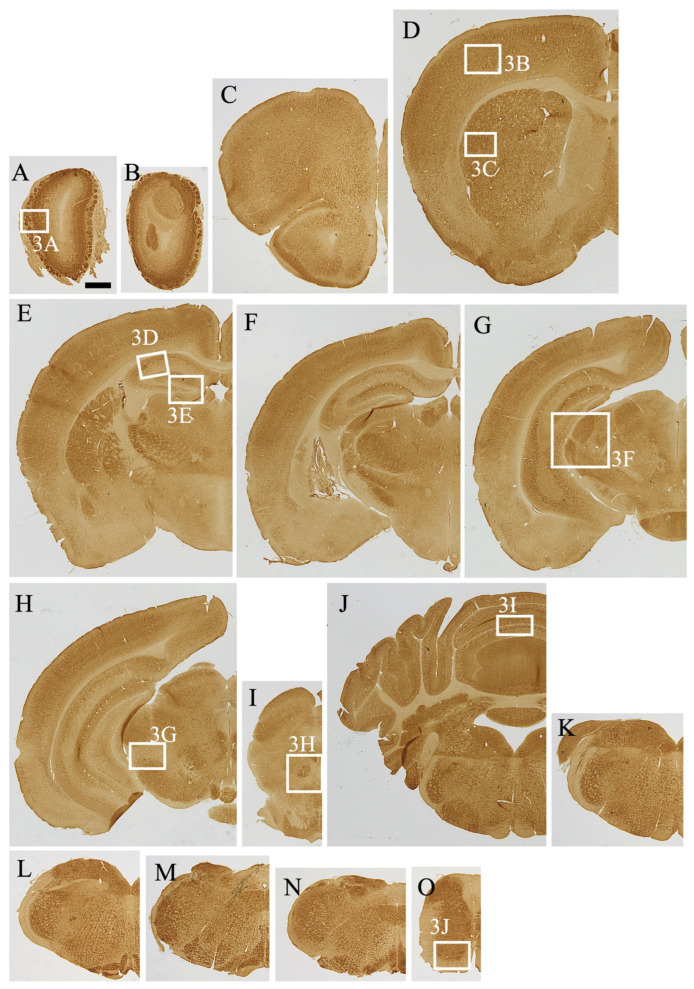
(**A**–**O**) MEGF8 immunoreactivity in the mouse central nervous system in the coronal section along the rostro-caudal axis. White boxes indicate the positions of the high-magnification images shown in Figure 3. Scale bar in (**A**) = 1 mm.

The distribution pattern of MEGF8 was investigated in the parasagittal mouse brain sections (Figure 1B). Immunohistochemical staining was used to determine the exact anatomical distribution of MEGF8 in the mouse brain sections (*n* = 4) (Figure 2). Strong MEGF8 expression was observed throughout the brain, including the olfactory bulb, cerebral cortex, hippocampus, basal ganglia, and cerebellar cortex (Figure 1B). 

MEGF8 IR (MEGF8-IR) was mainly observed in the gray matter, which had stronger MEGF8-IR than the white matter. In the gray matter, MEGF8-IR was observed in the neuropils surrounding the soma and neuronal somata.

### 3.3. Detailed Distribution of MEGF8-IR in the Mouse CNS

Table 1 displays MEGF8-IR in the CNS. The distribution patterns of MEGF8-IR were divided into two main types across various regions. The first type is located in the neuronal body, and the second is found in the neuropil. The second type is believed to be localized at synapses.

#### 3.3.1. Telencephalon

Olfactory bulb. A high intensity of MEGF8-IR was observed in the glomerular, mitral, and granular layers of the main olfactory bulb (Figure 2A). The MEGF8-IR intensity in the neuropils of the olfactory glomeruli was variable. The external plexiform layer showed moderate MEGF8-IR. Furthermore, moderate MEGF8-IR was observed in the vomeronasal neural layer and terminal regions of the accessory olfactory bulb.

Cerebral Cortex. MEGF8-IR showed a layer-specific pattern in all parts of the cerebral cortex (Figure 2B–G). Most neurons in the cerebral cortex were labeled with the anti-MEGF8 antibody. Notably, medium to large pyramidal neurons had high levels of MEGF8-IR. In comparison, neuropil staining was moderate in all layers.

**Figure 3:**
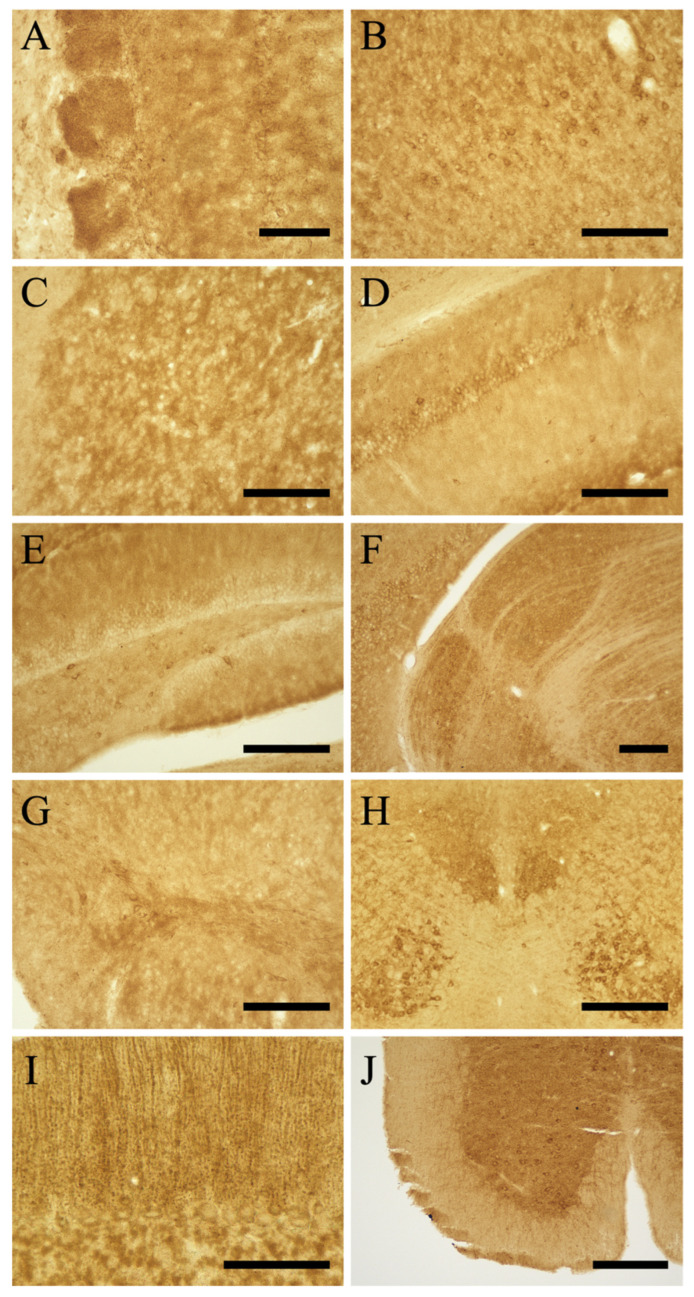
(**A**–**J**) High-magnification views of MEGF8 immunoreactivity shown in Figure 2. (**A**) olfactory bulb, (**B**) cerebral cortex, (**C**) caudate putamen, (**D**) Ammon’s horn of the hippocampus, (**E**) dentate gyrus, (**F**) dorsal and lateral geniculate nucleus, (**G**) lateral part of the substantia nigra, (**H**) dorsal raphe nucleus, (**I**) cerebellum, and (**J**) dorsal horn of the spinal cord. Scale bars in (**A**,**I**) = 100 μm, (**B**–**G**,**J**) = 200 μm, and (**H**) = 400 μm.

Hippocampus. Similar to the cerebral cortex, hippocampal cells exhibited layer-specific MEGF8-IR distribution. The CA1–3 pyramidal neuronal somata exhibited low to moderate MEGF8-IR (Figure 2D–G and Figure 3D). MEGF8-IR displayed moderate intensity in the CA1–2 radial and CA3 lucid layers. The dentate gyrus (DG) displayed strong staining, apart from the neuronal layer (Figure 2D–G and Figure 3D). The molecular layers of the DG showed high levels of neuropil staining. In comparison, the polymorphic layer (hilus) exhibited cell-specific and weak to moderate staining in the neuropil. In Ammon’s horn, moderate intensity of MEGF8-IR was observed in the neuropils of the stratum oriensis and stratum radiatum.

Fimbria and Corpus Callosum. Little to no MEGF8-IR was observed in the corpus callosum (Figure 2C–E). The majority of oligodendrocytes in the white matter did not express MEGF8. The subfornix organs exhibited a high intensity of MEGF8-IR in punctate cells.

Basal Forebrain and Septal Area. The caudate putamen gray matter (CPu) displayed relatively intense neuropil staining (Figure 2C and Figure 3C). The globus pallidus of the neuropil showed moderate MEGF8-IR levels. The nucleus accumbens core exhibited relatively high MEGF8-IR in neuropils (Figure 2C).

Olfactory Tubercle (Tu). Tu showed moderate to strong MEGF8-IR (Figure 2B,C). This staining pattern was consistent across the pyriform cortices of mice. The calleja islands and olfactory nuclei also exhibited moderate to strong MEGF8-IR. The lateral septal nucleus showed moderate MEGF8-IR. The medial septal nucleus showed moderate MEGF8 expression.

Amygdaloid Complex. All nuclei in the amygdala complex showed MEGF8-IR in neuropils. The central and basolateral nuclei exhibited moderate MEGF8-IR.

#### 3.3.2. Diencephalon

Hypothalamus. Numerous hypothalamic neurons expressed moderate to strong MEGF8-IR levels, particularly in the supraoptic, suprachiasmatic, magnocellular, and parvocellular regions of several subregions, including the paraventricular, arcuate, posterior hypothalamic, ventromedial hypothalamic, caudal, and posterior magnocellular nuclei (Figure 2G,K). The MEGF8-IR in the hypothalamus neuropils decreased laterally. The neuropils in the medial preoptic area, periventricular hypothalamus, and suprachiasmatic and arcuate nuclei exhibited moderate MEGF8-IR levels. MEGF8-IR was observed in both external and internal layers of the median eminence. Low MEGF8-IR levels were observed in zona incerta.

Thalamus. MEGF8-IR was observed in all thalamic nuclei. In particular, the medial habenula nucleus exhibited intense IR. In the medial and dorsolateral geniculate nuclei, intense MEGF8-IR was observed upon neuropil staining (Figure 2F,G). The paraventricular, medial habenula, medial geniculate, and ventral posterolateral nuclei showed moderate to strong MEGF8-IR levels in the neuropils. The olivary pronucleus exhibited a high staining intensity.

#### 3.3.3. Mesencephalon

Moderate to strong neuropil staining was observed in the superficial layers of the superior colliculus, gray periaqueduct, and interpeduncular nuclei. In the ventral tegmental region, MEGF8 showed a strong signal in the neuropil. In the red nuclei, large neurons showed strong MEGF8-IR and neuropil staining. 

#### 3.3.4. Pons and Medulla

Most neuropils displayed weak to moderate MEGF8-IR. The gray matter in these regions exhibited moderate to strong neuropil staining, whereas the white matter showed no staining.

At the pontine level, intense MEGF8-IR was observed in trapezoidal bodies. The locus coeruleus, motor trigeminal nucleus, principal sensory trigeminal nucleus, and dorsal raphe nucleus showed moderate to strong staining (Figure 2H,I). The retro-tubular area and pedunculopontine nuclei showed weak MEGF8-IR staining. In the inferior colliculus and periaqueductal gray, MEGF8-IR exhibited moderate to strong intensity in the neuropil. The mesencephalic trigeminal nucleus showed moderate MEGF8-IR. The trigeminal spinal cord and inferior olivary, laminar, and globus pallidum raphe nuclei showed moderate to strong MEGF8-IR in the neuropil. MEGF8-IR was intense in the dorsal cochlea, medial vestibular nucleus, ambiguous nucleus, and the dorsal motor nucleus of the vagus nerve. Furthermore, moderate to strong MEGF8-IR was detected in the ventral cochlea, facial nucleus, sublingual nucleus, and solitary tube nucleus. The reticular formation showed intense neuropil staining. The postrema also showed intense neuropil staining.

#### 3.3.5. Cerebellum

MEGF8-IR expression was observed in the cerebellum. In the molecular layer, intense MEGF8-IR was observed within the neuropil (Figure 2J). A moderate amount of MEGF8-IR was observed in the soma of the Purkinje and granule cells. The granular layer of the cerebellum showed strong staining of glomeruli, and several cerebellar nuclei were moderately stained.

#### 3.3.6. Spinal Cord

MEGF8-IR was observed throughout the spinal cord. The gray matter exhibited intense neuropil staining. In comparison, the white matter showed low staining, whereas strong MEGF8-IR staining was observed in the dorsal and ventral horns. Additionally, MEGF8-IR was observed in the cell bodies of the ventral horn.

### 3.4. Subcellular Localization of MEGF8

Immunoelectron microscopy was used to assess the subcellular localization of MEGF8-IR in neurons, with three samples analyzed for ultrastructural expression of MEGF8. We examined three parts of the brain: the cerebral cortex, hippocampus, and cerebellum.

#### 3.4.1. Cerebral Cortex

**Figure 4:**
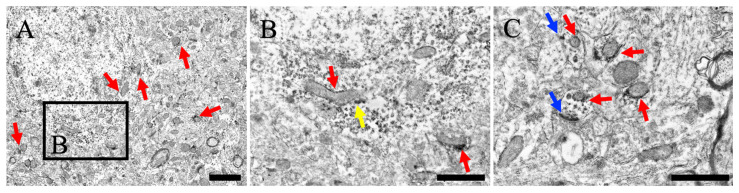
(**A**) Neurons and neuropils near the neuronal soma are present in layer V of the cerebral cortex. (**B**) Magnified view of (**A**). (**C**) The neuropil far from the neuronal soma. Red arrows indicate MEGF8 immunoreactivity near the mitochondria. Yellow arrows indicate MEGF8 immunoreactivity near subcellular organelles, without mitochondria. Blue arrows indicate MEGF8 immunoreactivity near the cell membrane, including the synapses. Scale bars: (**A**) = 2 μm; (**B**,**C**) = 1 μm.

**Figure 5:**
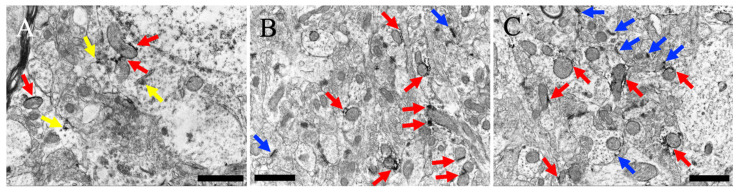
(**A**) Neurons and neuropils near the neuronal somata of the CA1 are shown. (**B**) Neuropil of CA1 far from the neuronal soma. (**C**) Neuropils in the dentate gyrus. Red arrows indicate MEGF8 immunoreactivity near the mitochondria. Yellow arrows indicate MEGF8 immunoreactivity near subcellular organelles, without mitochondria. Blue arrows indicate MEGF8 immunoreactivity near the cell membrane, including the synapses. All scale bars = 1 μm.

MEGF8-IR was observed within the neuronal perikarya in layer V of the cerebral cortex, close to the outer mitochondrial membrane (Figure 4A,B). Intracellular MEGF8 signals were detected in the endoplasmic reticulum and granules (Figure 5B). Additionally, MEGF8 was detected on the cell membrane, which was close to the membrane of post-synapses (Figure 4C).

#### 3.4.2. Hippocampus

MEGF8-IR was observed in neuronal cell somata in the CA1 region of the hippocampus, close to the mitochondrial cytoplasmic membrane (Figure 5A). MEGF8 signals were detected in the endoplasmic reticulum and granules (Figure 5A). MEGF8 signals were also detected in the CA1 neuropil, close to the mitochondria (Figure 5B), and in the cell membrane, including the synapses (Figure 5B). Similarly, in the DG, MEGF8 was found near the mitochondria (Figure 5C) and cell membranes, including the synapses (Figure 5C).

#### 3.4.3. Cerebellum

**Figure 6:**
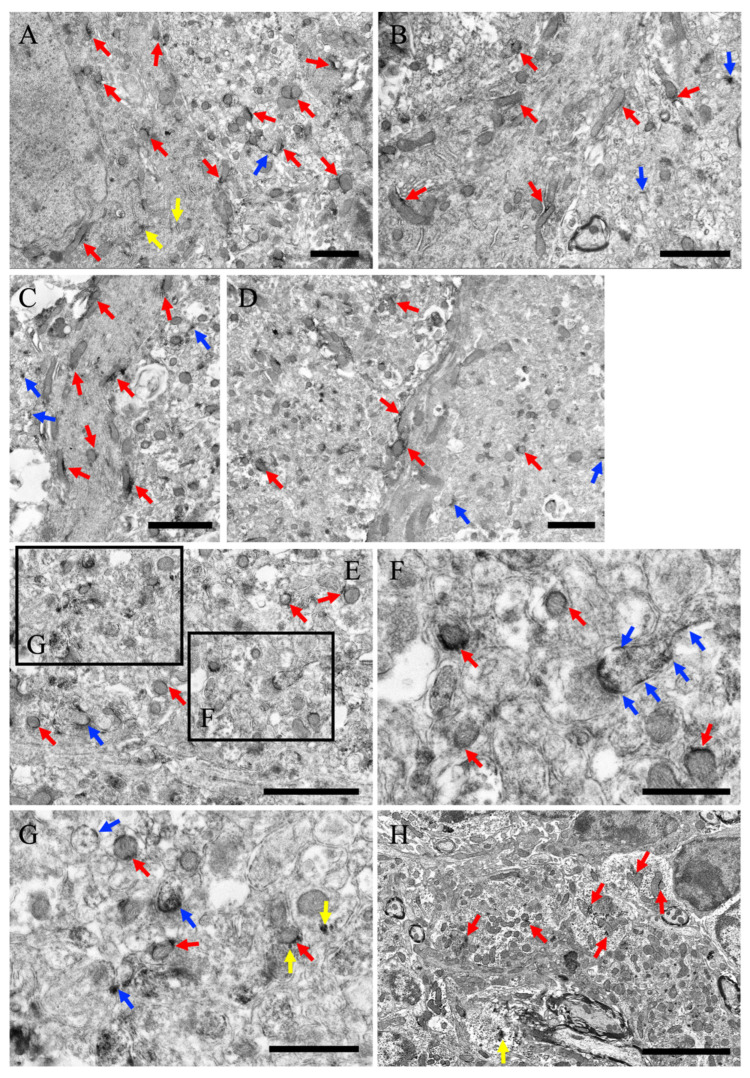
Immuno-electron microscopy of the cerebellum. (**A**) Purkinje cell somata and neuropils in the Purkinje cell layer. (**B**) Dendritic process (proximal portion) of Purkinje cells and the neuropil in the molecular layer. (**C**) Dendritic process (middle portion) of the Purkinje cells and the neuropil in the molecular layer. (**D**) Dendritic process (distal portion) of Purkinje cells and neuropils in the molecular layer. (**E**–**G**) The neuropil far from the neuronal soma. (**F**,**G**) Higher-magnification views of (**E**). (**H**) Granular somata and neuropils in the granular cell layer. Red arrows indicate MEGF8 immunoreactivity near the mitochondria. Yellow arrows indicate MEGF8 immunoreactivity near subcellular organelles, without mitochondria. Blue arrows indicate MEGF8 immunoreactivity near the cell membrane, including the synapses. Scale bars: (**A**–**E**) = 2 μm, (**F**,**G**) = 1 μm, and (**H**) = 5 μm.

Subsequently, we examined MEGF8 localization in the cerebellum of three individuals. MEGF8-IR was found close to the mitochondrial plasma membrane (Figure 6A) and other subcellular organelles (Figure 6A) in the Purkinje cell bodies of the Purkinje cell layer. Additionally, MEGF8 signals were detected in adjacent neuropils, most of which were located near the mitochondria, similar to those in the cell bodies (Figure 6A). MEGF8 was also observed in the cell membrane (Figure 6A). In the molecular layer, MEGF8 was expressed in the dendrites (distal to the apical part) of Purkinje cells, particularly close to the cytoplasmic membrane of the mitochondria (Figure 6B–D). Furthermore, MEGF8 was located close to the mitochondria (Figure 6B–G) and other subcellular organelles (Figure 6G) in neuropils. Moreover, MEGF8 was expressed on the cell membrane in close proximity to the postsynaptic membrane (Figure 6B–G). In the granular cell layer, strong MEGF8-IR was observed close to the mitochondria in the cerebellar glomerulus (Figure 6H). The MEGF8 protein expression patterns observed by electron microscopy corroborated the findings of the immunohistochemical analyses.

#### 3.4.4. Negative Control

**Figure 7:**
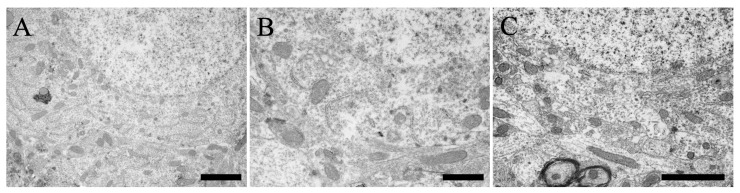
Immunoelectron microscopy of the cerebellum using a pre-adsorption antibody. (**A**) Neuron in layer V of the cerebral cortex. (**B**) Neuron in the CA1. (**C**) Purkinje cells in the Purkinje cell layer. Scale bars: (**A**,**C**) = 2 μm, and (**B**) = 1 μm.

The specificity of the antibody was validated through the pre-adsorption of the prepared antibody and peptide, followed by analysis of the resulting supernatant solution using immunoelectron microscopy (Figure 7). The results did not reveal notable signals in the cerebral cortex (Figure 7A), hippocampus (Figure 7B), and cerebellum (Figure 7C), suggesting the specificity of the signals observed in this study and efficacy of the antibodies utilized at the electron microscopy level.

## 4. Discussion

### 4.1. Specificity of MEGF8 Antibody

This study illustrated the distribution of MEGF8 in the CNS of mice. BLAST search results for the 16 amino acids used as antigens to generate antibodies in this experiment did not reveal any cross-reactivity with other proteins. Consequently, the antibodies produced in this study are thought to have a high degree of specificity. To assess the specificity of the anti-MEGF8 antibody in the mouse brain, immunoblotting and immunohistochemical analyses were performed. During immunoblotting, the anti-MEGF8 antibody identified a single band with a molecular weight of approximately 300 kDa, which was the predicted molecular weight of MEGF8 based on the amino acid sequence of mouse MEGF8 (GenBank Accession No. ACE00231.1). Immunohistochemical analysis revealed a distinct IR pattern of MEGF8. After pre-incubating the primary antibody with an abundance of each epitope peptide, MEGF8-IR was completely eliminated, indicating that the antibody specifically bound to MEGF8.

### 4.2. Distribution of MEGF8

Using the newly synthesized antibody, MEGF8-IR was found to be extensively distributed throughout the CNS of mice. Notably, moderate to strong MEGF8-IR was observed in many different regions, including the olfactory bulb, caudate putamen, nucleus accumbens, central and basolateral amygdaloid nuclei, medial preoptic area, paraventricular, arcuate, caudal and posterior magnocellular, paraventricular thalamic, ventral posterolateral, dorsal lateral geniculate, medial geniculate nuclei, superficial layer of the superior colliculus, interpeduncular nucleus, trapezoid body, dorsal and ventral cochlear nuclei, spinal trigeminal nucleus, raphe pallidus, nucleus ambiguus, dorsal column nuclei, and cerebellum. MEGF8-IR was also widely distributed in neuropils, particularly in the olfactory system (e.g., olfactory bulb and piriform cortex), visual system (e.g., dorsal lateral geniculate nucleus and superior colliculus), somatosensory system (e.g., spinal dorsal horn, trigeminal spinal nucleus, dorsal columnar nucleus, ventrolateral nucleus, and somatosensory system), and auditory and vestibular systems (e.g., cochlear nucleus, vestibular nucleus, and medial geniculate nucleus). In addition to the sensory system, MEGF8-IR was observed in motor systems, such as the somatic motor system (which includes the somatic motor cortex and ventral horn of the spinal cord). In this study, we examined the subcellular distribution of MEGF8 in the cerebral cortex (Figure 4), hippocampus (Figure 5), and cerebellum (Figure 6). The distribution of MEGF8 in the brain was similar to that of Atrn, which is believed to interact with MGRN1 and MEGF8 [14].

In all areas, MEGF8-IR was observed in the neuronal cell bodies and membranes, including synaptic terminals. Furthermore, immunoelectron microscopy data showed that the intracellular domain of MEGF8 was located in the cytoplasm close to the mitochondria (Figure 4, Figure 5 and Figure 6). The intracellular distribution of these components was similar to that of Atrn, which binds to MGRN1 in conjunction with MEGF8 [14]. Further research is needed to determine the role of MEGF8 in the sensory and motor systems; however, physiological studies in attractin-mutant rats or mice and MGRN1 mutant animals may help to uncover the potential roles of MEGF8 in these systems [33].

These findings suggest that MEGF8 is located close to the mitochondria in neurons and cell membranes, including synaptic terminals, across the CNS.

### 4.3. Relationship among the Attractin Family, MGRN1, and Spongiform Degeneration

Spongiform degeneration is characterized by the formation of vacuoles in neuronal tissues, accompanied by neuronal cell death and/or gliosis. Spongiform degeneration is a common symptom of neuropathological conditions such as Alzheimer’s disease [34], Canavan’s spongiform leukodystrophy [35,36], prion diseases [37], and HIV infection [38]. The similar spongiform degeneration observed in these diseases implies that the same intracellular mechanisms are responsible for spongiform changes and neuronal deterioration in the CNS. Abnormal ubiquitination can modify intracellular signaling and cell activity through both proteasome-dependent and proteasome-independent pathways, potentially resulting in spongiform degeneration and neuronal cell death.

The association between aberrant ubiquitination and corpus cavernosum neurodegeneration is further strengthened by the discovery that null mutations in the E3 ubiquitin protein ligase MGRN1 lead to an autosomal recessive form of corpus cavernosum neurodegeneration in animals [9]. *Mgrn1*- and *Atrn*-mutant mice display similar spongiform degeneration phenotypes, implying that MGRN1 and ATRN function via the same pathway. Recent studies support this hypothesis, as mice with no *Mgrn1* or *Atrn* genes demonstrate similar deficits in mitochondrial function and enhanced oxidative stress in brain proteins [39,40,41]. To date, no analyses of MEGF8 mutant mice have been published; however, MEGF8 deficiency in humans leads to mental retardation [1], which indicates that this leads to neuronal dysfunction. Our previous study demonstrated that ATRN is located close to the outer mitochondrial and cell membranes [14]. To the best of our knowledge, this is the first study to demonstrate MEGF8 protein expression in the CNS. Ultrastructural examination showed that the MEGF8 protein was located close to the mitochondrial outer membrane and cell membranes. Additionally, we previously demonstrated that MGRN1 has a shared binding motif for ATRN and MEGF8 in the central nervous system [13]. Furthermore, MGRN1 is found in pre-synapses and is in close proximity to the outer mitochondrial membrane. These findings indicate that signaling through MGRN1 and the UPS may be activated not only directly at the cell membrane, but also in the outer mitochondrial membrane. *Atrn* mutant mice displayed a significant decrease in the function of MGRN1 and the activity of cytochrome oxidase C [40]. Rodents with loss of ATRN and MGRN1 function display mitochondrial impairment, increased oxidative stress, and adult-onset spongiform neurodegeneration [9,42,43]. *ATRN* has been identified as a potential gene for sporadic amyotrophic lateral sclerosis in humans [44]. Its plasma levels change in individuals with early-onset Alzheimer’s disease before symptoms appear [45], and its gene expression is decreased in patients with Parkinson’s disease [46]. In terms of MEGF8, there have been no reports of neuronal damage in the brain. Nevertheless, both MEGF8 and ATRN utilize the same UPS, which is regulated by MGRN1. Consequently, future knockout experiments are expected to provide further details, such as similarities between the two as well as their individual effects and synergistic effects. According to these reports, it is highly probable that the ATRN/MEGF8-MGRN1 pathway is involved in the development of various neurological disorders. Nevertheless, it has been reported that the UPS, which employs other molecules, is also present in mitochondria [47,48,49]. This research indicates that the ATRN/MEGF8-MGRN1 pathway is present not only in the cell membrane, but also in the mitochondria.

In the present study, we investigated the intracerebral distribution of MEGF8, which binds to MGRN1, which in turn is involved in UPS. As further research is conducted and the pathways responsible for spongiform neurodegeneration are better understood, new and logical therapeutic strategies for treating prion diseases, HIV infection, and other spongiform degenerative diseases will be developed. This study elucidates the source of UPS regulated by MGRN1 in the brain, which has not been previously documented. These findings provide a foundation for further exploration of the mechanism of neuronal vacuolization and could aid in future examination of UPSs with analogous roles.

## 5. Conclusions

The study discusses the distribution of MEGF8, a transmembrane protein, in the mouse central nervous system (CNS). We used a new antibody to map MEGF8 in the CNS and found that it is distributed in the majority of the neuronal cell somata across most CNS regions, with high levels in the neuropils of the CNS gray matter. Immunoelectron microscopy showed that MEGF8 is present in the synapses and around the outer mitochondrial membrane. These findings suggest that MEGF8 plays a substantial role in the synaptic and mitochondrial functions in the CNS.

## Figures and Tables

**Table 1:** Distribution of MEGF8 in the mouse CNS.

Region					Immunoreactivity	Region				Immunoreactivity
Olfactory							Ventral posteromedial nucleus			++
	Main olfactory bulb						Dorsal lateral geniculate nucleus			+++
		Glomerular layer			++++		Intergeniculate leaflet			+
		External plexiform layer			+		Medial geniculate nucleus			+++
		Mitral layer			+++	Mesencephalon				
		Internal granular layer			+++		Superior colliculus			
	Accessory olfactory bulb				++			Superficial layer		+++
	Vomeronasal nerve layer				+			Deep layer		++
Cerebral cortex							Periaqueductal gray			+++
		Layer I–VI			++~++++		Oculomotor nucleus			++
Hippocampal formation							Red nucleus			+++
	Ammon’s horn (CA1–CA3)						Substantia nigra			+~+++
		Stratum oriens			++		Ventral tegmental area			+++
		Pyramidal layer			++		Interpeduncular nucleus			+++
		Stratum radiatum			+		Pons			
		Stratum lacunosum-moleculare			+		Dorsal tegmental nucleus			+++
	Dentate gyrus						Dorsal raphe nucleus			+~+++
		Molecular layer	Outside half		+++		Median raphe nucleus			++
			Inside half		+++		Parabrachial nucleus			++
		Granular layer			++		Inferior colliculus			++
		Hilus			++~+++		Retroauricular area			++
Basal forebrain and septal area							Pedunculopontine nucleus			++
	Bed nuclei of stria terminalis				++		Trapezoid body			+++
	Claustrum				++		Locus coeruleus			++
	Basal ganglia						Motor trigeminal nucleus			++
		Caudate putamen			+~++++		Principal sensory trigeminal nucleus			++
		Globus pallidus			+~++++	Medulla oblongata				
	Nucleus accumbens						Dorsal cochlear nucleus			+++
		Core			+++		Ventral cochlear nucleus			+++
		Shell			+~++		Medial vestibular nucleus			+++
	Olfactory tubercle				+~++		Other vestibular nuclei			++
	Island of Calleja				+++		Spinal trigeminal nucleus			+++
	Lateral septal nucleus				++		Facial nucleus			+++
	Medial septal nucleus				++		Raphe pallidus			++
	Nucleus of lateral olfactory tract				++		Ambiguus nucleus			+++
	Piriform cortex				+~+++		Nucleus of solitary tract			++
Amygdaloid complex							Dorsal motor nucleus of vagus			+++
	Central amygdaloid nucleus				++		hypoglossal nucleus			+++
	Basolateral amygdaloid nucleus				++		Inferior olive			++
	Medial amygdaloid nucleus				+++		External cuneate nucleus			+++
Hypothalamus							Cuneate nucleus			+++
	Medial preoptic area				+++		Gracile nucleus			+++
	Supraoptic nucleus				+++	Cerebellum				
	Suprachiasmatic nucleus				+++		Molecular layer			+++
	Paraventricular nucleus				+++		Purkinje cell layer			+~++
	Periventricular nucleus				+++		Granular cell layer			+~+++
	Lateral hypothalamic area				+~+++		Cerebellar nuclei			++
	Dorsal hypothalamic area				++	Spinal cord				
	Arcuate nucleus				++		Dorsal horn			+++
	Dorsomedial hypothalamic nucleus				++		Ventral horn			+++
	Ventromedial hypothalamic nucleus				+++	Circumventricular organ and related area				
	Zona incerta				+			Subfornical organ		+++
	Medial mammillary nucleus				++			Median eminence		+++
	Caudal and posterior magnocellular nuclei				++					
Epithalamus and thalamus								Subcommissural organ		+++
	Medial habenular nucleus				+++			Area postrema		+++
	Lateral habenular nucleus				++			Rostral migratory stream		++
	Paraventricular thalamic nucleus				+++			Ependyma		+
	Ventral posterolateral nucleus				++					

Expression of MEGF8 was evaluated by computer-associated densitometry: ++++, highest density; +++, higher density; ++, high density; +, low density; and ~, background density.

## Data Availability

Data are contained within the article.
